# Sleep, Pain, and Neurodegeneration: A Mendelian Randomization Study

**DOI:** 10.3389/fneur.2022.765321

**Published:** 2022-05-02

**Authors:** Sandeep Grover, Manu Sharma, International Age-related Macular Degeneration Genomics Consortium (IAMDGC)

**Affiliations:** Centre for Genetic Epidemiology, Institute for Clinical Epidemiology and Applied Biometry, University of Tübingen, Tübingen, Germany

**Keywords:** Mendelian randomization, causal inference, neurodegenerative disorders, sleep, pain, chronotype

## Abstract

Our aim was to determine whether the genetic liability to sleep and pain-related traits have a causal effect on risk of neurodegeneration in individuals of predominantly European ancestry. We selected five neurodegenerative disorders, namely, age-related macular degeneration (AMD), Alzheimer's disease (AD), amyotrophic lateral sclerosis (ALS), multiple sclerosis (MS), and Parkinson's disease (PD). Sleep duration (SD), short sleep (SS), long sleep (LS), chronotype (CHR), morning person (MP), insomnia (INS), and multisite chronic pain (MCP) were considered as exposures. We conducted Mendelian randomization (MR) using an inverse-variance weighted (IVW) method to compute causal effect estimates using latest available GWAS data sets. The MP phenotype was observed as the strongest risk factor for genetic liability to AMD (OR_IVW_ = 1.192; 95% CI 1.078, 1.318, *P* = 0.0007). We observed suggestive evidence of risky effects of CHR on AMD (*P* = 0.0034), SS on AD (*P* = 0.0044), and INS on ALS (*P* = 0.0123). However, we failed to observe any role of pain. The results were robust on sensitivity analyses. Our study highlighted the role of MP as a risk factor for AMD.

## Introduction

Patients with neurodegenerative diseases (NDDs) often experience disruptions in circadian rhythmic activities (1, 2). Many patients with NDD and circadian disruptions also complain of painful symptoms of variable origins and intensities (3). Both sleep and pain could often be treated and, thereby, can help maintain a stable quality of life in the absence of any disease-modifying treatment for NDDs (4). A greater understanding of the etiological relationship between sleep, pain, and neurodegeneration could, thereby, enable better management of NDDs.

It is well-recognized that circadian dysfunction in old age is due to degeneration of the suprachiasmatic nucleus (SCN) in the anterior hypothalamus, directly connected to the light-sensing retina (5). Different NDDs further exhibit marked heterogeneity in manifestation of circadian disruptions, which could be attributed to loss of different neuronal subpopulations in the SCN. Clinically, patients with Alzheimer's disease (AD) often show sleep-wake rhythm disorder, and patients with PD show a reduction in the amplitude of the circadian rhythm (6, 7). A limited number of longitudinal studies have demonstrated the potential influence of circadian disruptions on predisposition to AD, PD, and related markers of neurodegeneration (8, 9).

Similar to the involvement of specific brain regions directly influencing circadian rhythms, several brain regions, also referred to as pain matrix, have been shown to be activated during pain perception (10, 11). The pain matrix comprising the primary (S1) and secondary (S2) somatosensory cortices, insula, anterior cingulate cortex (ACC), amygdala, prefrontal cortex (PFC), and thalamus, further shows differential activation during acute and chronic pain (12). Aging is specifically known to increase the likelihood of chronic pain and may amplify the neurodegeneration process (13, 14).

To date, the sparse number of large longitudinal studies and clinical trials has limited our progress in understanding the relationship between sleep, pain, and onset or progression of neurodegeneration, necessitating the need for searching alternative approaches for judging the causality. A two-sample Mendelian randomization (MR) is one such approach that employs instruments or proxy markers of risk factor in one population to judge causality of the risk factor with an outcome in an independent population (15–17).

So far, limited studies have employed a genetic instrument-based approach to judge the etiological relationship between sleep, pain, and NDDs. A recent MR study showed an absence of the role of genetic liability to sleep duration (SD) in influencing predisposition to AD (18). On the contrary, another report showed an association of genetic liability with sleep efficiency with AD (19). A couple of studies showed increased risk of ALS due to daytime sleepiness (19, 20). Considering the highly varied role of various behavioral biomarkers of circadian rhythm on neurodegeneration and potential overlapping etiology of sleep and pain, we adopted a highly comprehensive approach by exploiting the availability of genetic instruments for various markers of circadian rhythm, namely, SD (21), short sleep (SS) (21), long sleep (LS) (21), chronotype (CHR) (22), morning person (MP) (22), insomnia (INS) (23), and multisite chronic pain (MCP) (24), and NDDs, namely, AD (25), AMD (26, 27), ALS (28), MS (29), and PD (30, 31) to dissect the bi-directional relationship between sleep, pain, and neurodegeneration using two-sample MR approach.

## Methods

### Identification and Correlation Among Data Sets

We employed a two-sample MR study design using summary estimates to examine the lifelong effect of sleep and pain-related traits on genetic liability to neurodegeneration in European populations. We used latest available discovery cohorts of meta-analyses of GWAS data sets in the literature. We identified single nucleotide polymorphisms (SNPs) that influence circadian rhythm-related traits, including SD (21), SS (21), LS (21), CHR (22), MP (22), INS (23), and MCP (24) (Table 1). We adopted a P cutoff of 5 × 10^−8^ to select the genetic instruments. Concerning the outcome data sets, we used the discovery cohort of a recent meta-analysis of GWAS on AD (25), AMD (26), ALS (28), MS (29), and PD (30). Before judging the causal role of sleep and pain in predisposition to PD, we checked for any potential correlation between different sleep and pain-related traits and different NDDs. We specifically employed a cross-trait LD score regression (LDSC) method to evaluate the genome-wide correlation between traits (https://github.com/bulik/ldsc) (32).

**Table 1:** Details of discovery GWAS datasets and prioritized instruments used for direct and reverse causal analysis in the present study.

**S.No**.	**Phenotype**	**References**	**Maximum sample size**	***P***	**Number of analyzed SNPs**	**Number of significant SNPs**	**Number of significant SNPs (post-clumping) (R^**2**^ < 0.001)**	**Average F-statistics Median (range)**	**R^**2**^ (%)**
**Sleep-related traits**									
1	Sleep duration (SD)	(21)	446,118	5 × 10^−8^	14,661,601	7,926	74	34.7 (29.6-220.9)	0.731%
2	Short sleep (SS)	(21)	106,192 cases/ 305,742 controls	5 × 10^−8^	14,661,601	859	26	34.1 (29.9-77.0)	0.045%
3	Long sleep (LS)	(21)	34,184 cases/ 305,742 controls	5 × 10^−8^	14,661,601	3,901	9	32.4 (29.9-53.0)	0.006%
4	Chronotype (CHR)	(22)	449,734	5 × 10^−8^	11,977,111	15,152	156	39.4 (28.2-209.4)	2.683%
5	Morning person (MP)	(22)	252,287 cases/ 150,908 controls	5 × 10^−8^	11,977,111	1,0949	127	37.9 (29.0-168.5)	5.748%
6	Insomnia (INS)	(23)	109,389 cases/ 277,144 controls	5 × 10^−8^	10,862,567	463	13	34.4 (30.4-94.7)	0.712%
**Pain-related trait**									
1	Mulisite chronic pain (MCP)	(24)	387,649	5 × 10^−8^	9,926,106	1,746	41	34.1 (30.0-54.6)	0.341%
**Disease trait**									
**Data sets used for main analysis**									
1	Alzheimer's disease (AD)	(25)	16,144 cases/ 17,832 controls	5 × 10^−8^	12,023,830	7,218	42	47.5 (29.2-382.5)	NA
2	Age-related macular degeneration (AMD)	(26)	71,880 cases/ 383,378 controls	5 × 10^−8^	3,367,299	2,357	27	42.2 (30.2-422.5)	NA
3	Amyotrophic lateral sclerosis (ALS)	(28)	12,577 cases/ 23,475 controls	5 × 10^−8^	8,709,452	125	4	37.2 (32.2-80.1)	NA
4	Multiple Sclerosis (MS)	(29)	47,351 cases/ 68,284 controls	5 × 10^−8^	8,593,650	26,403	74	41.9 (29.8-561.9)	NA
5	Parkinson's disease (PD)	(30)	33,674 cases, 449,056 controls	5 × 10^−8^	17,513,773	3,465	23	43.6 (30.0-181.5)	NA
**Data sets used for sensitivity analysis**									
1	Alzheimer's disease (AD) (without UKB)	(27)	17,008 cases/ 37,154 controls	5 × 10^−8^	7,055,881	1,090	18	37.9 (29.7-82.4)	NA
2	Parkinson's disease (PD) (without UKB)	(31)	9,581 cases/ 33,245 controls	5 × 10^−8^	8,543,957	3,209	9	49.8 (33.1-175.7)	NA

*Direct analysis was done using PD as an outcome and reverse was done using sleep and pain-related traits as outcomes*.

Since the study analyzed secondary data (publicly available data) that contained information at the population-level (summary-level data), informed consent and ethical approval were waived off for this study.

### Causal Effect Estimation

The prioritized SNP IDs and positions were synchronized with the NCBI GRCh37 assembly. We further checked for the validity of MR assumptions by excluding SNPs with F-statistics <10 and loci known to be directly involved in neurodegeneration based on existing evidence from previously published literature.

As the selected genetic instruments could be correlated, we performed clumping of significantly associated SNPs on each GWAS data set with the clump_data function of the TwoSampleMR package (version 0.4.25) in R (version 3.6.1). We employed a clumping window of 10,000 kb and linkage disequilibrium (LD; i.e., r^2^) cutoff of 0.001, and used the European population in the 1,000 Genome Phase 3v5 data set to identify the leading SNPs.

The leading SNPs were further checked for availability in the respective outcome data sets. When possible, if a specific SNP was not available, a proxy SNP (r^2^ > 0.8) was used. We further computed the pooled variance (R^2^) for the respective risk factor using effect estimates (β_x_) and effect allele frequencies (EAFs) of individual genetic instruments, i.e., R^2^ = 2*$\beta_{\text{x}}^{2}*$EAF*(1–EAF). Detectable risky and protective effect estimates at 80% power were computed for each NDD as an outcome at various pooled variances explained by the genetic instruments (ranging from 0.25 to 7.5%) using the Mendelian Randomisation Power Calculator (http://cnsgenomics.com/shiny/mRnd). To compute the effect estimates at specific variance for a given outcome, we employed a sample size of each outcome data set, the proportion of patients in the same data set, and a threshold *P* of 1.42 × 10^−3^.

We used the inverse variance-weighted (IVW) effect method as the primary method to compute the causal effect estimates, as used previously (17). We computed the causal estimates as odds ratio (OR) per unit of standard deviation (SD) for continuous traits and ORs for the outcome per unit log-odds of categorical traits. We employed a conservative Bonferroni correction of the significance level to account for 35 independent tests, including forward and reverse MR (threshold P = 1.42 × 10^−3^, i.e., 0.05/35). Heterogeneity was judged using the Cochran's Q-statistic and I^2^ for the IVW method along with Rucker's Q-statistic, and the intercept deviation test for the MR-Egger's method (17). All the scripts used for the primary MR analysis have been provided as part of the a R-based mrpipeline package (https://github.com/CGEatTuebingen/mrpipeline). We used a previously published data set to replicate the findings before employing the package for to this study (17). The mrpipeline package is currently under the developmental phase, with a plan to integrate external databases, including GWAS and tissue expression repositories in the future. We also performed an Mendelian Randomization Pleiotropy RESidual Sum and Outlier (MR-PRESSO) global test to evaluate horizontal pleiotropy (33). Lastly, we performed an MR Steiger test of directionality to validate the assumption that a given exposure causes an outcome using the TwoSampleMR package (version 0.4.25) in R (version 3.6.1).

### Sensitivity Analysis

Several approaches were employed to rule out the influence of potential pleiotropic variants on the overall results. We used multiple modern MR methods, including the MR-Egger, weighted median (WME), and weighted mode (MBE) methods, to check the reliability of the estimates, as used in previous studies (16, 17). Since most of the recent meta-analyses of GWAS compute effect estimates by pooling UK Biobank (UKB) data sets with previously available data sets, and the existence of any overlapping samples in exposure and outcome datasets could bias the effect estimates toward the confounded observational estimates, we also used the NDD datasets without UKB samples, when required (27, 31). We conducted MR in the reverse direction to check and confirm the directionality of the observed associations.

We further employed a leave-one-out and leave-one-group-out cross-validation approach to rule out the influence of outlier variants known to be associated with confounders of the relationship between the respective exposure and outcome data sets. We specifically employed the Phenoscanner database (http://phenoscanner.medschl.cam.ac.uk) to identify genetic variants associated with potential confounders. However, in the absence of knowledge of potential confounders, we adopted a more conservative approach, and all genetic loci known to be associated with non-sleep-related traits were assumed to be pleiotropic loci. We identified such loci by searching for all genetic variants in high LD with genetic instruments prioritized for this study using r^2^ > 0.9 for previously reported associations in European populations. We used visual approaches, including scatter plots and funnel plots, to identify outlier variants.

We also performed a sensitivity analysis by adjusting for potential confounders using a multivariable MR method. As and when appropriate, we adjusted for quantity of sleep, sleep preference for a given time of day, and pain, the phenotypes of interest investigated in this study. As multiple, highly correlated, and overlapping traits representing both quantity of sleep (LS, SS, INS, and SD) and sleep preference (CHR, MP) were available, we performed a variable selection procedure to select the optimal variable that represented each category. Such an approach prevented us from conducting an overadjustment and avoided loss of power inherent with multiple variable regression methods. We selected SD to adjust for the quantity of sleep, as a continuous variable is more informative than a binary trait. Similarly, we selected CHR as a variable of choice representing sleep preference. Specifically, the genetic associations of instruments with respective NDDs were regressed on the genetic associations with all the risk factors (SD, sleep pattern, and pain) in a single regression model using IVW method. Genetic instruments entered into the multivariable regression model were allowed to be associated with any of the risk factor under consideration.

We further evaluated the potential biological influence of different brain regions on their respective contribution to the causal effect estimate by analyzing gene expression data for available genetic variants from the Genotype-Tissue Expression Project (https://www.gtexportal.org).

## Results

### Identification and Correlation Among Data Sets

Details of discovery GWAS data sets used for the causal analysis in this study are shown in Table 1. The minimum number of individuals available for a specific NDD ranged from 12,557 ALS cases to 71,880 AD cases, which are broadly in consensus with their respective prevalence.

The pairwise genetic correlation analysis of complete GWAS data sets failed to show correlation of any of the NDDs with sleep or pain-related traits (Supplementary Table 1). Expectedly, a highly significant correlation was observed among the traits representing SD (SS, LS, SD, and INS) and among those representing sleep pattern (CHR and MP). Notably, MCP was strongly correlated with all the markers of SD (rg ranging from 0.28 for LS to 0.59 for INS), suggesting a need for conducting a multivariable analysis adjusting for MCP when judging the independent association of sleep markers with NDDs or vice versa.

### Causal Effect Estimation

The genetic instruments were identified that influence sleep and pain-related traits through latest publicly available meta-analysis of GWAS summary datasets (Table 1). Overall, we identified 771 genetic instruments to check the bidirectional causality between sleep, pain, and neurodegeneration, with F-statistic for individual SNPs ranging from 28.2 to 422.5. The detectable effect estimates for different NDDs as outcomes at 80% power and a type-1 error rate of 1.42 × 10^−3^ are further shown in Supplementary Table 2.

**Figure 1:**
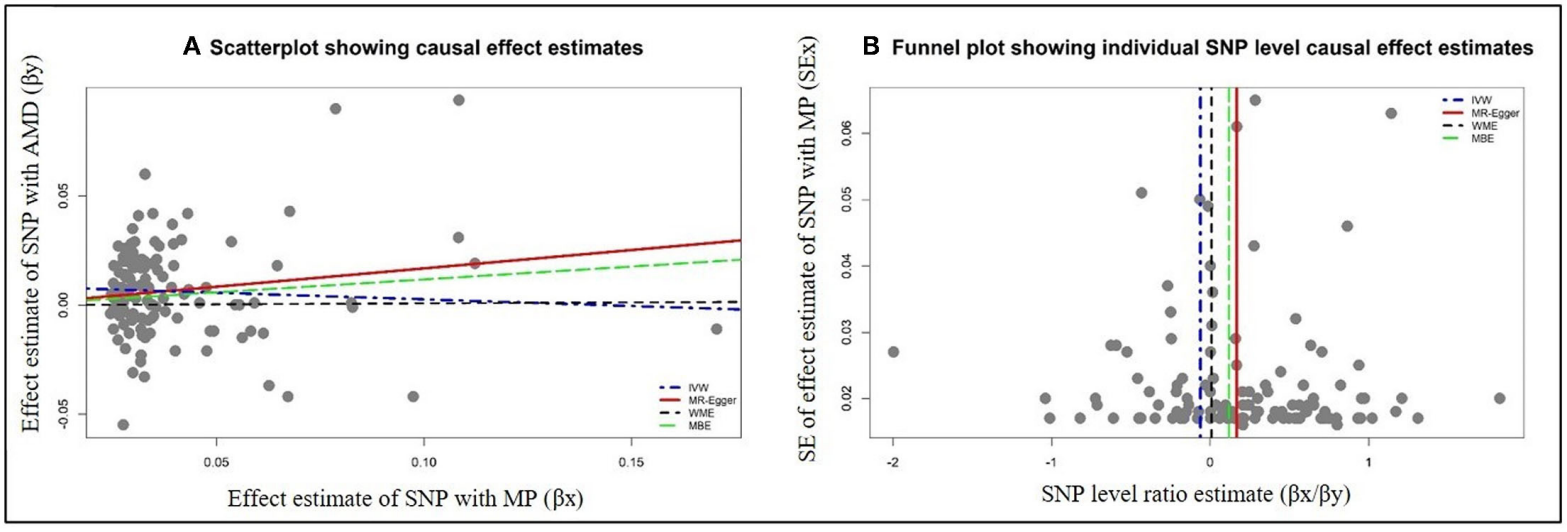
Graphical representation of causal association analysis and assessment of pleiotropy. **(A)** Scatterplot showing causal effect estimates computed using various MR methods for the association of morning person (MP) as exposure with age-related macular degeneration (AMD) as outcome. **(B)** Funnel plot showing the extent of heterogeneity among the individual Wald ratio estimates for morning person (MP) as exposure with age-related macular degeneration (AMD) as outcome. IVW, inverse variance-weighted method; WME, weighted median method; MBE, weighted mode method (NOME assumptions).

The data used for computation of causal effect estimates are provided in Supplementary Table 3. The causal effect estimates using various MR approaches and heterogeneity analysis measures used to judge the robustness of the estimates are provided in Table 2 for the direct causal estimates for NDDs as outcomes. We observed a highly significant causal effect of MP on genetic liability to AMD (OR_IVW_ = 1.192; 95% CI 1.078, 1.318, *P* = 0.0007). Heterogeneity check confirmed the reliability of the observed association with absence of any heterogeneity in the distribution of effect estimates of individual genetic variants (I^2^ = 0.0%, Cochran's *Q*-test *P* = 0.9288, Rucker's *Q*-test *P* = 0.9414, MR-PRESSO global test *P* = 0.8420). The distribution of individual SNP-level effect estimates and the effect estimates computed with different MR methods for the effect of MP on AMD is further shown as scatter and funnel plots in Figure 1. We observed a similar directionality of causal effect estimates using the WME method (OR_WME_ = 1.126; 95% CI = 1.044, 1.214). We also observed a similar trend using a highly correlated but continuous trait, CHR on AMD (OR_IVW_ = 1.269; 95% CI 1.083, 1.486, *P* = 0.0034). The directionality of findings was further confirmed by a significantly higher variance explained by genetic instruments for MP and CHR than that explained by the respective genetic instruments for AMD (P_Steiger_ = 2.1 × 10^−98^ and P_Steiger_ = 1.65 × 10^−24^). In contrast, we did not observe any direct role of pain on predisposition to AMD.

**Table 2:** Causal effect estimates using different Mendelian randomization (MR) methods and heterogeneity analysis of causal effect estimates for neurodegeneratice disorders (NDDs) using various sleep and pain-related traits as exposures.

**Trait**	**Mendelian randomization (MR) methodology**	**Number of SNPs**	**Direct causal effect estimates**			**Tests of heterogeneity**	
			**OR**	**95% CI**	***P***		
**Alzheimer's disease (AD)**							
Sleep duration (SD)	Inverse variance weighted method (IVW)	71	0.992	0.956-1.029	0.6567	MR-Egger intercept (*P*)	0.2022
	MR-Egger method		0.909	0.791-1.045	0.1783	I^2^ (IVW)	0.0%
	Weighted median method (WME)		0.998	0.971-1.026	0.9436	Cochran's Q-test (IVW) (P)	0.5815
	Weighted mode method (NOME		1.026	0.934-1.127	0.5951	Rucker's *Q*-test (*P*)	0.6021
	assumptions) (MBE)					Rucker's Q-test statistic/Cochran's Q-test statistic	0.9763
						MR-PRESSO global test (*P*)	0.4270
Short sleep (SS)	Inverse variance weighted method (IVW)	26	1.256	1.081-1.459	0.0044	MR-Egger intercept (*P*)	0.7405
	MR-Egger method		1.121	0.547-2.299	0.7457	I^2^ (IVW)	0.0%
	Weighted median method (WME)		1.219	1.103-1.347	0.0586	Cochran's Q-test (IVW) (P)	0.5847
	Weighted mode method (NOME		1.362	0.952-1.949	0.1032	Rucker's *Q*-test (*P*)	0.5279
	assumptions) (MBE)					Rucker's Q-test statistic/Cochran's Q-test statistic	0.9994
						MR-PRESSO global test (*P*)	0.4520
Long sleep (LS)	Inverse variance weighted method (IVW)	6	0.877	0.527-1.460	0.5381	MR-Egger intercept (*P*)	0.4714
	MR-Egger method		1.443	0.231-9.010	0.6076	I^2^ (IVW)	0.0%
	Weighted median method (WME)		0.979	0.763-1.255	0.9341	Cochran's Q-test (IVW) (P)	0.4411
	Weighted mode method (NOME		1.082	0.525-2.232	0.8385	Rucker's *Q*-test (*P*)	0.3854
	assumptions) (MBE)					Rucker's Q-test statistic/Cochran's Q-test statistic	0.8662
						MR-PRESSO global test (*P*)	0.4640
Chronotype (CHR)	Inverse variance weighted method (IVW)	153	0.995	0.973-1.018	0.6729	MR-Egger intercept (*P*)	0.0941
	MR-Egger method		0.937	0.871-1.009	0.0850	I^2^ (IVW)	28.6%
	Weighted median method (WME)		0.995	0.980-1.009	0.7090	Cochran's Q-test (IVW) (P)	0.0008
	Weighted mode method (NOME		1.025	0.934-1.125	0.6074	Rucker's *Q*-test (*P*)	0.0013
	assumptions) (MBE)					Rucker's Q-test statistic/Cochran's Q-test statistic	0.9810
						MR-PRESSO global test (*P*)	<0.001
Morning person (MP)	Inverse variance weighted method (IVW)	123	1.001	0.986-1.017	0.8441	MR-Egger intercept (*P*)	0.0364
	MR-Egger method		0.953	0.909-1.001	0.0533	I^2^ (IVW)	23.5%
	Weighted median method (WME)		1.004	0.994-1.014	0.7228	Cochran's Q-test (IVW) (P)	0.0127
	Weighted mode method (NOME		1.022	0.962-1.086	0.4776	Rucker's *Q*-test (*P*)	0.0234
	assumptions) (MBE)					Rucker's Q-test statistic/Cochran's Q-test statistic	0.9643
						MR-PRESSO global test (*P*)	0.0030
Insomnia (INS)	Inverse variance weighted method (IVW)	13	0.981	0.939-1.024	0.3448	MR-Egger intercept (*P*)	0.8399
	MR-Egger method		0.968	0.836-1.120	0.6342	I^2^ (IVW)	8.4%
	Weighted median method (WME)		0.977	0.953-1.001	0.3529	Cochran's Q-test (IVW) (P)	0.3621
	Weighted mode method (NOME		0.983	0.914-1.059	0.6655	Rucker's *Q*-test (*P*)	0.2882
	assumptions) (MBE)					Rucker's Q-test statistic/Cochran's Q-test statistic	0.9987
						MR-PRESSO global test (*P*)	0.2800
Multisite chronic pain (MCP)	Inverse variance weighted method (IVW)	32	1.373	0.884-2.133	0.1523	MR-Egger intercept (*P*)	0.0029
	MR-Egger method		25.956	3.919-171.909	0.0014	I^2^ (IVW)	13.1%
	Weighted median method (WME)		1.143	0.866-1.509	0.6338	Cochran's Q-test (IVW) (P)	0.2575
	Weighted mode method (NOME		1.026	0.305-3.457	0.9667	Rucker's *Q*-test (*P*)	0.6690
	assumptions) (MBE)					Rucker's Q-test statistic/Cochran's Q-test statistic	0.7320
						MR-PRESSO global test (*P*)	0.1730
**Age-related macular degeneration (AMD)**							
Sleep duration (SD)	Inverse variance weighted method (IVW)	69	1.242	0.925-1.667	0.1475	MR-Egger intercept (*P*)	0.0252
	MR-Egger method		0.397	0.141-1.117	0.0792	I^2^ (IVW)	0.0%
	Weighted median method (WME)		1.165	0.935-1.451	0.4888	Cochran's Q-test (IVW) (P)	0.5105
	Weighted mode method (NOME		1.198	0.546-2.629	0.6545	Rucker's *Q*-test (*P*)	0.6381
	assumptions) (MBE)					Rucker's Q-test statistic/Cochran's Q-test statistic	0.9302
						MR-PRESSO global test (*P*)	0.2830
Short sleep (SS)	Inverse variance weighted method (IVW)	25	0.520	0.144-1.881	0.3041	MR-Egger intercept (*P*)	0.8015
	MR-Egger method		0.249	0.001-113.336	0.6431	I^2^ (IVW)	10.0%
	Weighted median method (WME)		0.723	0.320-1.631	0.6936	Cochran's Q-test (IVW) (P)	0.3198
	Weighted mode method (NOME		0.967	0.050-18.823	0.9826	Rucker's *Q*-test (*P*)	0.2735
	assumptions) (MBE)					Rucker's Q-test statistic/Cochran's Q-test statistic	0.9970
						MR-PRESSO global test (*P*)	0.2210
Long sleep (LS)	Inverse variance weighted method (IVW)	6	1.355	0.004-491.772	0.8997	MR-Egger intercept (*P*)	0.7103
	MR-Egger method		41.952	NA	0.6982	I^2^ (IVW)	44.8%
	Weighted median method (WME)		2.690	0.321-22.563	0.6612	Cochran's Q-test (IVW) (P)	0.1066
	Weighted mode method (NOME		3.168	0.005-1904.134	0.7383	Rucker's *Q*-test (*P*)	0.0654
	assumptions) (MBE)					Rucker's Q-test statistic/Cochran's Q-test statistic	0.9745
						MR-PRESSO global test (*P*)	0.0730
Chronotype (CHR)	Inverse variance weighted method (IVW)	150	1.269	1.083-1.486	0.0034	MR-Egger intercept (*P*)	0.5248
	MR-Egger method		1.086	0.653-1.805	0.7503	I^2^ (IVW)	1.9%
	Weighted median method (WME)		1.171	1.048-1.308	0.1556	Cochran's Q-test (IVW) (P)	0.4204
	Weighted mode method (NOME		0.954	0.533-1.707	0.8736	Rucker's *Q*-test (*P*)	0.4104
	assumptions) (MBE)					Rucker's Q-test statistic/Cochran's Q-test statistic	0.9963
						MR-PRESSO global test (*P*)	0.0920
Morning person (MP)	Inverse variance weighted method (IVW)	121	1.192	1.078-1.318	0.0007	MR-Egger intercept (*P*)	0.1273
	MR-Egger method		0.941	0.682-1.297	0.7075	I^2^ (IVW)	0.0%
	Weighted median method (WME)		1.126	1.044-1.214	0.1197	Cochran's Q-test (IVW) (P)	0.9288
	Weighted mode method (NOME		1.008	0.682-1.491	0.9662	Rucker's *Q*-test (*P*)	0.9414
	assumptions) (MBE)					Rucker's Q-test statistic/Cochran's Q-test statistic	0.9771
						MR-PRESSO global test (*P*)	0.8420
Insomnia (INS)	Inverse variance weighted method (IVW)	13	1.135	0.826-1.560	0.4017	MR-Egger intercept (*P*)	0.2253
	MR-Egger method		2.158	0.686-6.793	0.1678	I^2^ (IVW)	0.0%
	Weighted median method (WME)		1.113	0.927-1.336	0.5694	Cochran's Q-test (IVW) (P)	0.8587
	Weighted mode method (NOME		1.120	0.622-2.016	0.7119	Rucker's *Q*-test (*P*)	0.9109
	assumptions) (MBE)					Rucker's Q-test statistic/Cochran's Q-test statistic	0.7715
						MR-PRESSO global test (*P*)	0.8530
Multisite chronic pain (MCP)	Inverse variance weighted method (IVW)	31	1.014	0.580-1.774	0.9597	MR-Egger intercept (*P*)	0.1034
	MR-Egger method		0.120	0.009-1.702	0.1129	I^2^ (IVW)	3.6%
	Weighted median method (WME)		1.279	0.897-1.825	0.4931	Cochran's Q-test (IVW) (P)	0.4092
	Weighted mode method (NOME		1.250	0.303-5.158	0.7594	Rucker's *Q*-test (*P*)	0.5018
	assumptions) (MBE)					Rucker's Q-test statistic/Cochran's Q-test statistic	0.9093
						MR-PRESSO global test (*P*)	0.2860
**Amyotrophic lateral sclerosis (ALS)**							
Sleep duration (SD)	Inverse variance weighted method (IVW)	71	1.003	0.743-1.355	0.9844	MR-Egger intercept (*P*)	0.2855
	MR-Egger method		0.569	0.191-1.696	0.3069	I^2^ (IVW)	0.0%
	Weighted median method (WME)		1.011	0.797-1.283	0.9621	Cochran's Q-test (IVW) (P)	0.5246
	Weighted mode method (NOME		0.988	0.410-2.384	0.9795	Rucker's *Q*-test (*P*)	0.5307
	assumptions) (MBE)					Rucker's Q-test statistic/Cochran's Q-test statistic	0.9829
						MR-PRESSO global test (*P*)	0.3920
Short sleep (SS)	Inverse variance weighted method (IVW)	26	0.839	0.231-3.052	0.7818	MR-Egger intercept (*P*)	0.7964
	MR-Egger method		1.837	0.003-1038.801	0.8447	I^2^ (IVW)	4.7%
	Weighted median method (WME)		0.693	0.296-1.624	0.6705	Cochran's Q-test (IVW) (P)	0.3947
	Weighted mode method (NOME		0.587	0.022-15.815	0.7541	Rucker's *Q*-test (*P*)	0.3451
	assumptions) (MBE)					Rucker's Q-test statistic/Cochran's Q-test statistic	0.9969
						MR-PRESSO global test (*P*)	0.3180
Long sleep (LS)	Inverse variance weighted method (IVW)	6	0.746	0.003-218.829	0.8994	MR-Egger intercept (*P*)	0.8766
	MR-Egger method		0.223	NA	0.8550	I^2^ (IVW)	40.2%
	Weighted median method (WME)		0.505	0.060-4.219	0.7606	Cochrane Q-test (IVW) (*P*)	0.1375
	Weighted mode method (NOME		0.350	0.001-242.154	0.7659	Rucker's Q-test (*P*)	0.0814
	assumptions) (MBE)					Rucker's test statistic/ Cochrane Q-statistic	0.9919
						MR-PRESSO global test (*P*)	0.1370
Chronotype (CHR)	Inverse variance weighted method (IVW)	153	0.914	0.781-1.070	0.2605	MR-Egger intercept (*P*)	0.8658
	MR-Egger method		0.876	0.524-1.467	0.6134	I^2^ (IVW)	0.0%
	Weighted median method (WME)		0.976	0.868-1.097	0.8343	Cochran's Q-test (IVW) (P)	0.5552
	Weighted mode method (NOME		1.112	0.642-1.924	0.7058	Rucker's *Q*-test (*P*)	0.5325
	assumptions) (MBE)					Rucker's Q-test statistic/Cochran's Q-test statistic	1.0000
						MR-PRESSO global test (*P*)	0.2740
Morning person (MP)	Inverse variance weighted method (IVW)	122	0.934	0.841-1.037	0.2007	MR-Egger intercept (*P*)	0.9094
	MR-Egger method		0.952	0.674-1.344	0.7779	I^2^ (IVW)	0.0%
	Weighted median method (WME)		0.944	0.873-1.020	0.4607	Cochran's Q-test (IVW) (P)	0.8461
	Weighted mode method (NOME		1.012	0.711-1.439	0.9480	Rucker's *Q*-test (*P*)	0.8302
	assumptions) (MBE)					Rucker's Q-test statistic/Cochran's Q-test statistic	0.9998
						MR-PRESSO global test (*P*)	0.7370
Insomnia (INS)	Inverse variance weighted method (IVW)	13	1.551	1.121-2.145	0.0123	MR-Egger intercept (*P*)	0.4410
	MR-Egger method		1.100	0.404-2.993	0.8383	I^2^ (IVW)	0.0%
	Weighted median method (WME)		1.480	1.203-1.821	0.0828	Cochran's Q-test (IVW) (P)	0.5894
	Weighted mode method (NOME		1.386	0.762-2.522	0.3063	Rucker's *Q*-test (*P*)	0.5559
	assumptions) (MBE)					Rucker's Q-test statistic/Cochran's Q-test statistic	0.9432
						MR-PRESSO global test (*P*)	0.5290
Multisite chronic pain (MCP)	Inverse variance weighted method (IVW)	35	1.472	0.902-2.401	0.1176	MR-Egger intercept (*P*)	0.3001
	MR-Egger method		0.412	0.034-5.066	0.4772	I^2^ (IVW)	16.8%
	Weighted median method (WME)		1.456	1.085-1.954	0.2097	Cochran's Q-test (IVW) (P)	0.1938
	Weighted mode method (NOME		1.586	0.484-5.195	0.4512	Rucker's *Q*-test (*P*)	0.1943
	assumptions) (MBE)					Rucker's Q-test statistic/Cochran's Q-test statistic	0.9726
						MR-PRESSO global test (*P*)	0.0740
**Multiple sclerosis (MS)**							
Sleep duration (SD)	Inverse variance weighted method (IVW)	70	1.002	0.732-1.371	0.9909	MR-Egger intercept (*P*)	0.2162
	MR-Egger method		2.014	0.632-6.423	0.2323	I^2^ (IVW)	9.3%
	Weighted median method (WME)		1.133	0.911-1.408	0.5684	Cochran's Q-test (IVW) (P)	0.2622
	Weighted mode method (NOME assumptions) (MBE)		1.168	0.581-2.346	0.6641	Rucker's *Q*-test (*P*)	0.2822
						Rucker's Q-test statistic/Cochran's Q-test statistic	0.9763
						MR-PRESSO global test (*P*)	0.0810
Short sleep (SS)	Inverse variance weighted method (IVW)	26	4.780	0.939-24.326	0.0588	MR-Egger intercept (*P*)	0.8463
	MR-Egger method		10.264	NA	0.5641	I^2^ (IVW)	42.7%
	Weighted median method (WME)		1.740	0.732-4.137	0.5284	Cochran's Q-test (IVW) (P)	0.0120
	Weighted mode method (NOME		0.724	0.046-11.380	0.8199	Rucker's *Q*-test (*P*)	0.0083
	assumptions) (MBE)					Rucker's Q-test statistic/Cochran's Q-test statistic	1.0017
						MR-PRESSO global test (*P*)	0.0010
Long sleep (LS)	Inverse variance weighted method (IVW)	5	0.296	0.001-90.815	0.5866	MR-Egger intercept (*P*)	0.1757
	MR-Egger method		NA	NA	0.2011	I^2^ (IVW)	28.5%
	Weighted median method (WME)		4.452	0.479-41.384	0.5397	Cochran's Q-test (IVW) (P)	0.2318
	Weighted mode method (NOME		5.847	0.016-2101.701	0.5880	Rucker's *Q*-test (*P*)	0.4244
	assumptions) (MBE)					Rucker's Q-test statistic/Cochran's Q-test statistic	0.4997
						MR-PRESSO global test (*P*)	0.2190
Chronotype (CHR)	Inverse variance weighted method (IVW)	154	1.022	0.715-1.461	0.9041	MR-Egger intercept (*P*)	0.2977
	MR-Egger method		0.553	0.164-1.863	0.3370	I^2^ (IVW)	30.1%
	Weighted median method (WME)		0.940	0.836-1.055	0.5928	Cochran's Q-test (IVW) (P)	0.0004
	Weighted mode method (NOME assumptions) (MBE)		0.853	0.467-1.558	0.6058	Rucker's *Q*-test (*P*)	0.0001
	assumptions) (MBE)					Rucker's test statistic/Cochrane *Q*-statistic	1.0236
						MR-PRESSO global test (*P*)	<0.001
Morning person (MP)	Inverse variance weighted method (IVW)	124	0.963	0.856-1.084	0.5337	MR-Egger intercept (*P*)	0.4641
	MR-Egger method		0.833	0.554-1.254	0.3788	I^2^ (IVW)	22.3%
	Weighted median method (WME)		0.964	0.894-1.039	0.6234	Cochran's Q-test (IVW) (P)	0.0176
	Weighted mode method (NOME		0.931	0.634-1.367	0.7172	Rucker's *Q*-test (*P*)	0.0167
	assumptions) (MBE)					Rucker's Q-test statistic/Cochran's Q-test statistic	0.9954
						MR-PRESSO global test (*P*)	0.0010
Insomnia (INS)	Inverse variance weighted method (IVW)	13	0.936	0.648-1.352	0.7029	MR-Egger intercept (*P*)	0.5462
	MR-Egger method		0.658	0.179-2.422	0.4944	I^2^ (IVW)	19.9%
	Weighted median method (WME)		0.850	0.696-1.038	0.4327	Cochran's Q-test (IVW) (P)	0.2429
	Weighted mode method (NOME		0.859	0.495-1.489	0.5974	Rucker's *Q*-test (*P*)	0.2091
	assumptions) (MBE)					Rucker's Q-test statistic/Cochran's Q-test statistic	0.9650
						MR-PRESSO global test (*P*)	0.1920
Multisite chronic pain (MCP)	Inverse variance weighted method (IVW)	34	1.444	0.861-2.422	0.1577	MR-Egger intercept (*P*)	0.9268
	MR-Egger method		1.635	0.101-26.412	0.7212	I^2^ (IVW)	28.7%
	Weighted median method (WME)		1.197	0.890-1.609	0.5483	Cochran's Q-test (IVW) (P)	0.0619
	Weighted mode method (NOME		1.237	0.417-3.668	0.7038	Rucker's *Q*-test (*P*)	0.0483
	assumptions) (MBE)					Rucker's Q-test statistic/Cochran's Q-test statistic	1.0010
						MR-PRESSO global test (*P*)	0.0220
**Parkinson's disease (PD)**							
Sleep duration (SD)	Inverse variance weighted method (IVW)	70	0.934	0.649-1.343	0.7085	MR-Egger intercept (*P*)	0.3304
	MR-Egger method		0.475	0.115-1.970	0.3003	I^2^ (IVW)	6.3%
	Weighted median method (WME)		0.805	0.626-1.034	0.3889	Cochran's Q-test (IVW) (P)	0.3284
	Weighted mode method (NOME		0.652	0.244-1.743	0.3968	Rucker's *Q*-test (*P*)	0.3251
	assumptions) (MBE)					Rucker's Q-test statistic/Cochran's Q-test statistic	0.9874
						MR-PRESSO global test (*P*)	0.1550
Short sleep (SS)	Inverse variance weighted method (IVW)	26	3.485	0.810-14.993	0.0903	MR-Egger intercept (*P*)	0.8351
	MR-Egger method		1.742	0.002-1841.723	0.8708	I^2^ (IVW)	0.0%
	Weighted median method (WME)		2.734	1.025-7.290	0.3149	Cochran's Q-test (IVW) (P)	0.4655
	Weighted mode method (NOME		3.892	0.113-133.950	0.4587	Rucker's *Q*-test (*P*)	0.4079
	assumptions) (MBE)					Rucker's Q-test statistic/Cochran's Q-test statistic	1.0006
						MR-PRESSO global test (*P*)	0.2820
Long sleep (LS)	Inverse variance weighted method (IVW)	6	0.506	0.002-121.424	0.7627	MR-Egger intercept (*P*)	0.1399
	MR-Egger method		0.000	0.000-158.569	0.1383	I^2^ (IVW)	17.0%
	Weighted median method (WME)		0.075	0.006-0.938	0.3522	Cochran's Q-test (IVW) (P)	0.3036
	Weighted mode method (NOME		0.019	0.000-37.008	0.3521	Rucker's *Q*-test (*P*)	0.5936
	assumptions) (MBE)					Rucker's Q-test statistic/Cochran's Q-test statistic	0.4629
						MR-PRESSO global test (*P*)	0.2590
Chronotype (CHR)	Inverse variance weighted method (IVW)	155	0.921	0.753-1.125	0.4158	MR-Egger intercept (*P*)	0.5143
	MR-Egger method		1.116	0.603-2.065	0.7250	I^2^ (IVW)	20.9%
	Weighted median method (WME)		0.875	0.763-1.003	0.3280	Cochran's Q-test (IVW) (P)	0.0149
	Weighted mode method (NOME		0.805	0.496-1.308	0.3823	Rucker's *Q*-test (*P*)	0.0141
	assumptions) (MBE)					Rucker's Q-test statistic/Cochran's Q-test statistic	0.9966
						MR-PRESSO global test (*P*)	<0.001
Morning person (MP)	Inverse variance weighted method (IVW)	125	1.026	0.898-1.173	0.7011	MR-Egger intercept (*P*)	0.8819
	MR-Egger method		0.996	0.658-1.509	0.9863	I^2^ (IVW)	15.2%
	Weighted median method (WME)		0.938	0.858-1.024	0.4666	Cochran's Q-test (IVW) (P)	0.0847
	Weighted mode method (NOME		0.903	0.650-1.254	0.5446	Rucker's *Q*-test (*P*)	0.0754
	assumptions) (MBE)					Rucker's Q-test statistic/Cochran's Q-test statistic	1.0001
						MR-PRESSO global test (*P*)	0.0080
Insomnia (INS)	Inverse variance weighted method (IVW)	13	1.100	0.692-1.747	0.6609	MR-Egger intercept (*P*)	0.4819
	MR-Egger method		0.628	0.108-3.657	0.5726	I^2^ (IVW)	34.1%
	Weighted median method (WME)		0.891	0.694-1.146	0.6549	Cochran's Q-test (IVW) (P)	0.1093
	Weighted mode method (NOME		0.654	0.278-1.539	0.3500	Rucker's *Q*-test (*P*)	0.0953
	assumptions) (MBE)					Rucker's Q-test statistic/Cochran's Q-test statistic	0.9578
						MR-PRESSO global test (*P*)	0.1730
Multisite chronic pain (MCP)	Inverse variance weighted method (IVW)	34	0.696	0.435-1.113	0.1259	MR-Egger intercept (*P*)	0.2551
	MR-Egger method		0.200	0.021-1.876	0.1531	I^2^ (IVW)	0.0%
	Weighted median method (WME)		0.728	0.532-0.995	0.3184	Cochran's Q-test (IVW) (P)	0.6387
	Weighted mode method (NOME		0.545	0.163-1.8118	0.3304	Rucker's *Q*-test (*P*)	0.6541
	assumptions) (MBE)					Rucker's Q-test statistic/Cochran's Q-test statistic	0.9573
						MR-PRESSO global test (*P*)	0.5230

We further observed a suggestive risky causal effect of SS on genetic liability to AD (OR_IVW_ = 1.256; 95% CI 1.081, 1.459, *P* = 0.0044). Heterogeneity check further confirmed the reliability of the observed association with absence of any heterogeneity in the distribution of effect estimates of individual genetic variants (I^2^ = 0%, Cochrane *P* =0.5847, Rucker's *Q*-test *P* = 0.5279, MR-PRESSO global test *P* = 0.4270). A similar directionality in the causal effect estimates was also observed using the WME method (OR = 1.121; 95% CI 1.103, 1.347). However, we did not observe any role of pain in predisposition to AD.

We also observed a suggestive risky causal effect of INS on genetic liability to ALS (OR_IVW_ = 1.551; 95% CI 1.121, 2.145, *P* = 0.0123). On the other hand, we failed to observe any role of pain in predisposition to ALS.

We did not observe any direct role of sleep and pain-related traits in predisposition to MS. Similarly, our MR analysis failed to detect a role of the sleep and pain-related traits in predisposition to PD.

### Sensitivity Analysis

Concerning direct MR, the association of SS with AD was lost after the exclusion of overlapping UKB samples (data not shown). In the reverse MR, PD showed suggestion of a strong protective effect against CHR and MP after the exclusion of overlapping UKB samples (data not shown). Reverse causal estimates for various sleep and pain-related traits using various NDDs as exposure are shown in Table 3. Our reverse casual check confirmed the directionality of the observed associations of MP and CHR with AMD, as we failed to observe any effect of AMD on MP and CHR. Our reverse causal check also confirmed the role of SS in predisposition to AD, as we failed to observe the causal effect of AD on SS. Interestingly, all the sleep-related traits except for SS were observed to be influenced by genetic predisposition to AD when employing non-IVW methods for judging causal effects of sleep-related traits on AD. Lastly, our reverse casual check confirmed the role of INS in predisposition to ALS. On the contrary, our findings suggested a causal role of genetic predisposition to ALS in LS with a consistent significant risk effect using the IVW, WME, and MBE methods.

**Table 3:** Causal effect estimates using different Mendelian randomization methods and heterogeneity analysis of causal effect estimates for various sleep and pain-related traits using Neurodegenerative disorders (NDDs) asexposures.

**Trait**	**MR methodology**	**Number of SNPs**	**Reverse causal effect estimates**			**Tests of heterogeneity**	
			**β or OR**	**95% CI**	***P***		
**Alzheimer's disease (AD)**							
Sleep duration (SD)	Inverse variance weighted method (IVW)	26	−0.0370	−0.0879-0.0140	0.1482	MR-Egger intercept (*P*)	0.0857
	MR-Egger method		−0.1046	−0.1977-−0.0114	0.0293	I^2^ (IVW)	0.0%
	Weighted median method (WME)		−0.0583	−0.0924-−0.0242	0.0996	Cochran's Q-test (IVW) (P)	0.6942
	Weighted mode method (NOME		−0.0854	−0.1644-−0.0064	0.0443	Rucker's *Q*-test (*P*)	0.8057
	assumptions) (MBE)					Rucker's Q-test statistic/Cochran's Q-test statistic	0.8557
						MR-PRESSO global test (*P*)	0.5860
Short sleep (SS)	Inverse variance weighted method (IVW)	26	1.004	0.983-1.026	0.6774	MR-Egger intercept (*P*)	0.0745
	MR-Egger method		1.035	0.995-1.076	0.0862	I^2^ (IVW)	0.0%
	Weighted median method (WME)		1.023	1.008-1.038	0.1287	Cochran's Q-test (IVW) (P)	0.9298
	Weighted mode method (NOME		1.026	0.993-1.060	0.1416	Rucker's *Q*-test (*P*)	0.9787
	assumptions) (MBE)					Rucker's Q-test statistic/Cochran's Q-test statistic	0.7827
						MR-PRESSO global test (*P*)	0.9120
Long sleep (LS)	Inverse variance weighted method (IVW)	26	0.984	0.968-1.000	0.0479	MR-Egger intercept (*P*)	0.9637
	MR-Egger method		0.984	0.955-1.014	0.2819	I^2^ (IVW)	0.0%
	Weighted median method (WME)		0.986	0.975-0.998	0.2373	Cochran's Q-test (IVW) (P)	0.7454
	Weighted mode method (NOME		0.973	0.944-1.002	0.0841	Rucker's *Q*-test (*P*)	0.6947
	assumptions) (MBE)					Rucker's Q-test statistic/Cochran's Q-test statistic	1.0005
						MR-PRESSO global test (*P*)	0.6330
Chronotype (CHR)	Inverse variance weighted method (IVW)	26	1.033	0.950-1.123	0.4365	MR-Egger intercept (*P*)	0.3102
	MR-Egger method		1.101	0.945-1.284	0.2055	I^2^ (IVW)	42.8%
	Weighted median method (WME)		1.123	1.075-1.173	0.0141	Cochran's Q-test (IVW) (P)	0.0118
	Weighted mode method (NOME		1.118	1.016-1.230	0.0306	Rucker's *Q*-test (*P*)	0.0158
	assumptions) (MBE)					Rucker's Q-test statistic/Cochran's Q-test statistic	0.9434
						MR-PRESSO global test (*P*)	0.0030
Morning person (MP)	Inverse variance weighted method (IVW)	26	1.055	0.922-1.205	0.4212	MR-Egger intercept (*P*)	0.5355
	MR-Egger method		1.123	0.877-1.438	0.3423	I^2^ (IVW)	0.4%
	Weighted median method (WME)		1.169	1.088-1.256	0.0388	Cochrane Q-test (IVW) (*P*)	0.0324
	Weighted mode method (NOME		1.177	1.003-1.381	0.0570	Rucker's *Q*-test (*P*)	0.0300
	assumptions) (MBE)					Rucker's Q-test statistic/Cochran's Q-test statistic	0.976
						MR-PRESSO global test (*P*)	0.0110
Insomnia (INS)	Inverse variance weighted method (IVW)	26	0.916	0.799-1.051	0.2011	MR-Egger intercept (*P*)	0.0599
	MR-Egger method		0.757	0.596-0.960	0.0239	I^2^ (IVW)	23.7%
	Weighted median method (WME)		0.871	0.799-0.948	0.1171	Cochran's Q-test (IVW) (P)	0.1372
	Weighted mode method (NOME		0.851	0.714-1.017	0.0888	Rucker's *Q*-test (*P*)	0.2441
	assumptions) (MBE)					Rucker's Q-test statistic/Cochran's Q-test statistic	0.8665
						MR-PRESSO global test (*P*)	0.0920
Multisite chronic pain (MCP)	Inverse variance weighted method (IVW)	26	−0.0371	−0.1073-0.0329	0.3181	MR-Egger intercept (*P*)	0.3786
	MR-Egger method		−0.0839	−0.2125-0.0447	0.1908	I^2^ (IVW)	33.2%
	Weighted median method (WME)		−0.0294	−0.0688-0.0100	0.4629	Cochran's Q-test (IVW) (P)	0.0527
	Weighted mode method (NOME		−0.0837	−0.1884-0.0209	0.1293	Rucker's *Q*-test (*P*)	0.0575
	assumptions) (MBE)					Rucker's Q-test statistic/Cochran's Q-test statistic	0.9566
						MR-PRESSO global test (*P*)	0.0140
**Amyotrophic lateral sclerosis (ALS)**							
Sleep duration (SD)	Inverse variance weighted method (IVW)	4	0.0249	−0.0054-0.0554	0.0797	MR-Egger intercept (*P*)	0.8095
	MR-Egger method		0.0816	−0.0963-0.1327	0.5654	I^2^ (IVW)	0.0%
	Weighted median method (WME)		0.0250	0.0138-0.03611	0.1099	Cochran's Q-test (IVW) (P)	0.9179
	Weighted mode method (NOME		0.0255	−0.0010-0.0521	0.1565	Rucker's *Q*-test (*P*)	0.803
	assumptions) (MBE)					Rucker's Q-test statistic/Cochran's Q-test statistic	0.8697
						MR-PRESSO global test (*P*)	0.9350
Short sleep (SS)	Inverse variance weighted method (IVW)	4	0.9980	0.985-1.011	0.6380	MR-Egger intercept (*P*)	0.9973
	MR-Egger method		0.9980	0.951-1.048	0.8711	I^2^ (IVW)	0.0%
	Weighted median method (WME)		0.9990	0.995-1.004	0.9524	Cochran's Q-test (IVW) (P)	0.7351
	Weighted mode method (NOME		1.0070	0.990-1.012	0.9091	Rucker's *Q*-test (*P*)	0.5285
	assumptions) (MBE)					Rucker's Q-test statistic/Cochran's Q-test statistic	1.002
						MR-PRESSO global test (*P*)	0.6800
Long sleep (LS)	Inverse variance weighted method (IVW)	4	1.0125	1.002-1.023	0.0316	MR-Egger intercept (*P*)	0.8888
	MR-Egger method		1.0109	0.964-1.059	0.4300	I^2^ (IVW)	4.0%
	Weighted median method (WME)		1.0134	1.009-1.017	0.0409	Cochran's Q-test (IVW) (P)	0.373
	Weighted mode method (NOME		1.0139	1.005-1.022	0.0530	Rucker's *Q*-test (*P*)	0.2079
	assumptions) (MBE)					Rucker's Q-test statistic/Cochran's Q-test statistic	1.0058
						MR-PRESSO global test (*P*)	0.4410
Chronotype (CHR)	Inverse variance weighted method (IVW)	4	1.0263	0.990-1.064	0.1068	MR-Egger intercept (*P*)	0.5384
	MR-Egger method		1.0488	0.915-1.202	0.2709	I^2^ (IVW)	0.0%
	Weighted median method (WME)		1.0294	1.016-1.043	0.1207	Cochran's Q-test (IVW) (P)	0.7821
	Weighted mode method (NOME		1.0328	1.001-1.065	0.1339	Rucker's *Q*-test (*P*)	0.7597
	assumptions) (MBE)					Rucker's Q-test statistic/Cochran's Q-test statistic	0.5093
						MR-PRESSO global test (*P*)	0.7830
Morning person (MP)	Inverse variance weighted method (IVW)	4	1.0383	0.977-1.103	0.1433	MR-Egger intercept (*P*)	0.5715
	MR-Egger method		1.0733	0.854-1.348	0.3137	I^2^ (IVW)	0.0%
	Weighted median method (WME)		1.0483	1.024-1.073	0.1327	Cochran's Q-test (IVW) (P)	0.6704
	Weighted mode method (NOME		1.0536	1.001-1.109	0.1397	Rucker's *Q*-test (*P*)	0.5750
	assumptions) (MBE)					Rucker's Q-test statistic/Cochran's Q-test statistic	0.7132
						MR-PRESSO global test (*P*)	0.6050
Insomnia (INS)	Inverse variance weighted method (IVW)	4	1.0148	0.947-1.087	0.5445	MR-Egger intercept (*P*)	0.9715
	MR-Egger method		1.0125	0.782-1.311	0.8555	I^2^ (IVW)	0.0%
	Weighted median method (WME)		1.0245	0.999-1.051	0.4104	Cochran's Q-test (IVW) (P)	0.6546
	Weighted mode method (NOME		1.0326	0.974-1.095	0.3607	Rucker's *Q*-test (*P*)	0.4449
	assumptions) (MBE)					Rucker's Q-test statistic/Cochran's Q-test statistic	0.9989
						MR-PRESSO global test (*P*)	0.6330
Multisite chronic pain (MCP)	Inverse variance weighted method (IVW)	4	0.0045	−0.0280-0.0372	0.6848	MR-Egger intercept (*P*)	0.5017
	MR-Egger method		−0.0169	−0.13931-0.1054	0.6112	I^2^ (IVW)	0.0%
	Weighted median method (WME)		0.0050	−0.0069-0.0169	0.7024	Cochran's Q-test (IVW) (P)	0.5233
	Weighted mode method (NOME		−0.0055	−0.0326-0.0216	0.7177	Rucker's *Q*-test (*P*)	0.4402
						Rucker's Q-test statistic/Cochran's Q-test statistic	0.7313
						MR-PRESSO global test (*P*)	0.4220
**Age related macular degeneration (AMD)**							
Sleep duration (SD)	Inverse variance weighted method (IVW)	38	−0.0005	−0.0077-0.0067	0.8752	MR-Egger intercept (*P*)	0.6714
	MR-Egger method		−0.0026	−0.0151-0.0099	0.6746	I^2^ (IVW)	47.3%
	Weighted median method (WME)		0.0014	−0.0022-0.0051	0.7060	Cochran's Q-test (IVW) (P)	0.0008
	Weighted mode method (NOME		0.0018	−0.0067-0.0103	0.6725	Rucker's *Q*-test (*P*)	0.0006
	assumptions) (MBE)					Rucker's Q-test statistic/Cochran's Q-test statistic	0.9966
						MR-PRESSO global test (*P*)	<0.001
Short sleep (SS)	Inverse variance weighted method (IVW)	38	0.999	0.997-1.001	0.2404	MR-Egger intercept (*P*)	0.7791
	MR-Egger method		0.999	0.995-1.003	0.6506	I^2^ (IVW)	6.5%
	Weighted median method (WME)		0.999	0.997-1.000	0.3791	Cochran's Q-test (IVW) (P)	0.3558
	Weighted mode method (NOME		0.997	0.993-1.001	0.1737	Rucker's *Q*-test (*P*)	0.3151
	assumptions) (MBE)					Rucker's Q-test statistic/Cochran's Q-test statistic	0.999
						MR-PRESSO global test (*P*)	0.2540
Long sleep (LS)	Inverse variance weighted method (IVW)	38	0.999	0.996-1.001	0.3476	MR-Egger intercept (*P*)	0.4268
	MR-Egger method		0.997	0.993-1.002	0.2361	I^2^ (IVW)	55.4%
	Weighted median method (WME)		1.000	0.999-1.001	0.9789	Cochran's Q-test (IVW) (P)	<0.0001
	Weighted mode method (NOME		1.001	0.997-1.004	0.6871	Rucker's *Q*-test (*P*)	<0.0001
	assumptions) (MBE)					Rucker's Q-test statistic/Cochran's Q-test statistic	0.9767
						MR-PRESSO global test (*P*)	<0.001
Chronotype (CHR)	Inverse variance weighted method (IVW)	38	1.005	0.994-1.015	0.3798	MR-Egger intercept (*P*)	0.6299
	MR-Egger method		1.001	0.983-1.019	0.9068	I^2^ (IVW)	63.8%
	Weighted median method (WME)		1.004	0.999-1.009	0.4304	Cochran's Q-test (IVW) (P)	<0.0001
	Weighted mode method (NOME		1.000	0.986-1.014	0.9980	Rucker's *Q*-test (*P*)	<0.0001
	assumptions) (MBE)					Rucker's Q-test statistic/Cochran's Q-test statistic	1.003
						MR-PRESSO global test (*P*)	<0.001
Morning person (MP)	Inverse variance weighted method (IVW)	38	1.007	0.991-1.025	0.3592	MR-Egger intercept (*P*)	0.5705
	MR-Egger method		1.001	0.973-1.030	0.9438	I^2^ (IVW)	58.3%
	Weighted median method (WME)		1.007	0.999-1.016	0.4027	Cochran's Q-test (IVW) (P)	<0.0001
	Weighted mode method (NOME		1.005	0.983-1.028	0.6701	Rucker's *Q*-test (*P*)	<0.0001
	assumptions) (MBE)					Rucker's Q-test statistic/Cochran's Q-test statistic	1.0019
						MR-PRESSO global test (*P*)	<0.001
Insomnia (INS)	Inverse variance weighted method (IVW)	37	0.994	0.981-1.008	0.3903	MR-Egger intercept (*P*)	0.4319
	MR-Egger method		0.987	0.965-1.010	0.2580	I^2^ (IVW)	27.6%
	Weighted median method (WME)		1.000	0.991-1.009	0.9879	Rucker's *Q*-test (*P*)	0.0656
	Weighted mode method (NOME		1.005	0.980-1.032	0.6813	Rucker's *Q*-test (*P*)	0.0614
	assumptions) (MBE)					Rucker's Q-test statistic/Cochran's Q-test statistic	0.9833
						MR-PRESSO global test (*P*)	0.0280
Multisite chronic pain (MCP)	Inverse variance weighted method (IVW)	42	−0.0028	−0.0089-0.0033	0.3574	MR-Egger intercept (*P*)	0.0548
	MR-Egger method		−0.0112	−0.0216-−0.0008	0.0358	I^2^ (IVW)	30.8%
	Weighted median method (WME)		−0.0034	−0.0075-0.0008	0.4127	Cochran's Q-test (IVW) (P)	0.0321
	Weighted mode method (NOME		−0.0021	−0.0119-0.0077	0.6812	Rucker's *Q*-test (*P*)	0.0686
	assumptions) (MBE)					Rucker's Q-test statistic/Cochran's Q-test statistic	0.911
						MR-PRESSO global test (*P*)	0.0110
**Multiple sclerosis (MS)**							
Sleep duration (SD)	Inverse variance weighted method (IVW)	70	0.0032	−0.0024-0.0088	0.2586	MR-Egger intercept (*P*)	0.6248
	MR-Egger method		0.0015	−0.0076-0.0105	0.7477	I^2^ (IVW)	53.2%
	Weighted median method (WME)		0.0044	0.0012-0.0076	0.1841	Cochran's Q-test (IVW) (P)	<0.0001
	Weighted mode method (NOME		0.0038	−0.0021-0.0097	0.2164	Rucker's *Q*-test (*P*)	<0.0001
	assumptions) (MBE)					Rucker's Q-test statistic/Cochran's Q-test statistic	0.9996
						MR-PRESSO global test (*P*)	NA
Short sleep (SS)	Inverse variance weighted method (IVW)	70	1.000	0.998-1.002	0.9521	MR-Egger intercept (*P*)	0.7509
	MR-Egger method		1.000	0.997-1.004	0.8329	I^2^ (IVW)	49.7%
	Weighted median method (WME)		0.999	0.997-1.000	0.4187	Cochran's Q-test (IVW) (P)	<0.0001
	Weighted mode method (NOME		0.999	0.997-1.002	0.6820	Rucker's *Q*-test (*P*)	<0.0001
	assumptions) (MBE)					Rucker's Q-test statistic/Cochran's Q-test statistic	0.9997
						MR-PRESSO global test (*P*)	NA
Long sleep (LS)	Inverse variance weighted method (IVW)	70	1.002	1.001-1.003	0.0040	MR-Egger intercept (*P*)	0.6275
	MR-Egger method		1.001	1.000-1.003	0.1459	I^2^ (IVW)	0.8%
	Weighted median method (WME)		1.002	1.001-1.003	0.1231	Cochran's Q-test (IVW) (P)	0.4591
	Weighted mode method (NOME		1.002	1.000-1.003	0.0726	Rucker's *Q*-test (*P*)	0.4326
	assumptions) (MBE)					Rucker's Q-test statistic/Cochran's Q-test statistic	0.9969
						MR-PRESSO global test (*P*)	NA
Chronotype (CHR)	Inverse variance weighted method (IVW)	70	1.003	0.996-1.009	0.3943	MR-Egger intercept (*P*)	0.2149
	MR-Egger method		0.998	0.987-1.008	0.6608	I^2^ (IVW)	52.3%
	Weighted median method (WME)		1.000	0.997-1.004	0.9476	Cochran's Q-test (IVW) (P)	<0.0001
	Weighted mode method (NOME		1.002	0.996-1.008	0.5352	Rucker's *Q*-test (*P*)	<0.0001
	assumptions) (MBE)					Rucker's Q-test statistic/Cochran's Q-test statistic	0.9815
						MR-PRESSO global test (*P*)	NA
Morning person (MP)	Inverse variance weighted method (IVW)	70	1.004	0.993-1.014	0.4939	MR-Egger intercept (*P*)	0.1581
	MR-Egger method		0.994	0.978-1.011	0.4975	I^2^ (IVW)	49.4%
	Weighted median method (WME)		0.998	0.992-1.004	0.7365	Cochran's Q-test (IVW) (P)	<0.0001
	Weighted mode method (NOME		1.002	0.992-1.013	0.6579	Rucker's *Q*-test (*P*)	<0.0001
	assumptions) (MBE)					Rucker's Q-test statistic/Cochran's Q-test statistic	0.973
						MR-PRESSO global test (*P*)	NA
Insomnia (INS)	Inverse variance weighted method (IVW)	67	1.000	0.991-1.011	0.8216	MR-Egger intercept (*P*)	0.5171
	MR-Egger method		1.005	0.989-1.021	0.5208	I^2^ (IVW)	27.6%
	Weighted median method (WME)		0.999	0.991-1.007	0.8708	Cochran's Q-test (IVW) (P)	0.0217
	Weighted mode method (NOME		1.003	0.990-1.015	0.6794	Rucker's *Q*-test (*P*)	0.0190
	assumptions) (MBE)					Rucker's Q-test statistic/Cochran's Q-test statistic	0.9956
						MR-PRESSO global test (*P*)	NA
Multisite chronic pain (MCP)	Inverse variance weighted method (IVW)	70	−0.0008	−0.0060-0.0043	0.6868	MR-Egger intercept (*P*)	0.7972
	MR-Egger method		−0.0017	−0.0100-0.0067	0.6902	I^2^ (IVW)	39.0%
	Weighted median method (WME)		−0.0032	−0.0066-0.0001	0.3276	Cochran's Q-test (IVW) (P)	0.0006
	Weighted mode method (NOME		−0.0043	−0.0100-0.0013	0.1428	Rucker's *Q*-test (*P*)	0.0005
	assumptions) (MBE)					Rucker's Q-test statistic/Cochran's Q-test statistic	0.9992
						MR-PRESSO global test (*P*)	NA
**Parkinson's disease (PD)**							
Sleep duration (SD)	Inverse variance weighted method (IVW)	23	0.0098	−0.0048-0.0245	0.1798	MR-Egger intercept (*P*)	0.93
	MR-Egger method		0.0113	−0.0266-0.0492	0.5417	I^2^ (IVW)	68.2%
	Weighted median method (WME)		0.0061	−0.0001-0.0125	0.3089	Cochran's Q-test (IVW) (P)	<0.0001
	Weighted mode method (NOME		−0.0001	−0.0193-0.0190	0.9877	Rucker's *Q*-test (*P*)	<0.0001
	assumptions) (MBE)					Rucker's Q-test statistic/Cochran's Q-test statistic	1.0032
						MR-PRESSO global test (*P*)	<0.001
Short sleep (SS)	Inverse variance weighted method (IVW)	23	0.999	0.995-1.002	0.3680	MR-Egger intercept (*P*)	0.8565
	MR-Egger method		0.998	0.988-1.007	0.6047	I^2^ (IVW)	19.0%
	Weighted median method (WME)		0.999	0.997-1.001	0.6722	Cochran's Q-test (IVW) (P)	0.2058
	Weighted mode method (NOME		1.002	0.993-1.010	0.6937	Rucker's *Q*-test (*P*)	0.1678
	assumptions) (MBE)					Rucker's Q-test statistic/Cochran's Q-test statistic	0.9982
						MR-PRESSO global test (*P*)	0.1170
Long sleep (LS)	Inverse variance weighted method (IVW)	23	1.002	0.998-1.007	0.2488	MR-Egger intercept (*P*)	0.7717
	MR-Egger method		1.004	0.993-1.016	0.4337	Cochran's Q-test (IVW) (P)	61.8%
	Weighted median method (WME)		0.999	0.997-1.001	0.4874	Rucker's *Q*-test (*P*)	<0.0001
	Weighted mode method (NOME		0.998	0.994-1.002	0.3944	Rucker's Q-test (*P*)	<0.0001
	assumptions) (MBE)					Rucker's Q-test statistic/Cochran's Q-test statistic	1.0081
						MR-PRESSO global test (*P*)	<0.001
Chronotype (CHR)	Inverse variance weighted method (IVW)	23	0.992	0.978-1.007	0.3039	MR-Egger intercept (*P*)	0.4215
	MR-Egger method		0.979	0.943-1.017	0.2560	I^2^ (IVW)	60.6%
	Weighted median method (WME)		1.002	0.994-1.009	0.8195	Cochran's Q-test (IVW) (P)	0.0001
	Weighted mode method (NOME		1.013	0.956-1.072	0.6693	Rucker's *Q*-test (*P*)	0.0001
	assumptions) (MBE)					Rucker's Q-test statistic/Cochran's Q-test statistic	0.9688
						MR-PRESSO global test (*P*)	<0.001
Morning person (MP)	Inverse variance weighted method (IVW)	23	0.991	0.967-1.015	0.4437	MR-Egger intercept (*P*)	0.5548
	MR-Egger method		0.974	0.915-1.038	0.4014	I^2^ (IVW)	60.0%
	Weighted median method (WME)		0.998	0.985-1.011	0.8945	Cochran's Q-test (IVW) (P)	0.0001
	Weighted mode method (NOME		0.943	0.875-1.016	0.1385	Rucker's *Q*-test (*P*)	0.0001
	assumptions) (MBE)					Rucker's Q-test statistic/Cochran's Q-test statistic	0.9819
						MR-PRESSO global test (*P*)	<0.001
Insomnia (INS)	Inverse variance weighted method (IVW)	23	1.002	0.980-1.024	0.8525	MR-Egger intercept (*P*)	0.8117
	MR-Egger method		0.996	0.942-1.053	0.8829	I^2^ (IVW)	34.8%
	Weighted median method (WME)		0.991	0.979-1.004	0.5141	Cochran's Q-test (IVW) (P)	0.0524
	Weighted mode method (NOME		0.967	0.931-1.004	0.0898	Rucker's *Q*-test (*P*)	0.0398
	assumptions) (MBE)					Rucker's Q-test statistic/Cochran's Q-test statistic	0.9967
						MR-PRESSO global test (*P*)	0.0240
Multisite chronic pain (MCP)	Inverse variance weighted method (IVW)	23	−0.0054	−0.0170-0.0062	0.3590	MR-Egger intercept (*P*)	0.3476
	MR-Egger method		−0.0178	−0.0472-0.0115	0.2202	I^2^ (IVW)	48.3%
	Weighted median method (WME)		−0.0093	−0.0155-−0.0032	0.1373	Cochran's Q-test (IVW) (P)	0.0054
	Weighted mode method (NOME		−0.0105	−0.0274-0.0062	0.2277	Rucker's *Q*-test (*P*)	0.0061
	assumptions) (MBE)					Rucker's Q-test statistic/Cochran's Q-test statistic	0.9565
						MR-PRESSO global test (*P*)	0.001

We failed to observe the predominant influence of any of the single variants on causal the effect estimates of MP with AMD, as shown in Supplementary Table 4. Similarly, the observed associations of CHR with AMD, SS with AD, and INS with ALS were retained (Supplementary Table 5). Among SNPs used for causal effect estimation of MP and CHR with AMD, 46 and 51 were identified as potential pleitropic variants for respective estimations (Supplementary Table 6). However, exclusion of these SNPs did not influence the observed casual association of MP and CHR with AMD (OR = 1.202, 95% CI 1.055, 1.370; OR = 1.262, 95% CI 1.049, 1.520). On the contrary, associations of SS with AD and INS with ALS were lost, which could be attributed to the presence of a high proportion of pleiotropic SNPs in the genetic instruments for SS and INS.

The sensitivity analysis using the multivariable MR approach also yielded similar results with the retention of the association of MP and CHR with AMD (OR = 1.184, 95% CI 1.083, 1.284; OR = 1.162, 95% CI 1.060, 1.263) (Supplementary Table 7).

Concerning the influence of specific brain regions, we specifically identified a high proportion of SNPs influencing brain expression in the cerebellum and basal ganglia region (Table 4). However, exclusion of these SNPs did not affect the overall causal association of CHR and MP with AMD. Similarly, we failed to observe the effect of any of the other brain regions on the observed associations. We also failed to observe any influence of brain region-specific expression on other observed associations (data not shown).

**Table 4:** Sensitivity analysis of causal effect estimates of sleep-related traits on neurodegeneration by exploring potential influence of specific brain region using variants involved in regional expression.

	**Causal effect estimates of MP with AMD**					**Causal effect estimates of CHR with AMD**				
**Brain region**	**Number of SNPs involved in expression**	**Number of SNPs remaining**	**IVW OR**	**95% CI**	***P***	**Number of SNPs involved in expression**	**Number of SNPs**	**IVW OR**	**95% CI**	***P***
Amygdala	5	116	1.184	1.069-1.312	0.0014	6	144	1.245	1.061-1.462	0.0077
Anterior cingulate cortex (BA24)	8	113	1.188	1.070-1.318	0.0014	12	138	1.269	1.088-1.479	0.0027
Brain—caudate (basal ganglia)	14	107	1.180	1.061-1.313	0.0027	19	131	1.262	1.075-1.482	0.0049
Brain—Cerebellar Hemisphere	13	108	1.185	1.066-1.317	0.0019	17	133	1.285	1.098-1.504	0.0020
Brain—cerebellum	16	105	1.186	1.065-1.320	0.0021	21	129	1.271	1.079-1.497	0.0044
Brain—cortex	13	108	1.175	1.058-1.306	0.0030	17	133	1.252	1.071-1.462	0.0050
Brain—cerebellar hemisphere	13	108	1.185	1.066-1.317	0.0019	17	133	1.285	1.098-1.504	0.0020
Brain—frontal cortex (BA9)	14	107	1.176	1.057-1.307	0.0031	15	135	1.264	1.083-1.476	0.0033
Brain—hippocampus	7	114	1.197	1.080-1.328	0.0008	11	139	1.296	1.111-1.511	0.0011
Brain—hypothalamus brain	0	121	1.192	1.078-1.318	0.0007	0	150	1.269	1.083-1.486	0.0034
Brain—nucleus accumbens (basal ganglia)	12	109	1.189	1.070-1.320	0.0015	17	133	1.268	1.082-1.486	0.0037
Brain—putamen (basal ganglia)	8	113	1.195	1.078-1.326	0.0009	12	138	1.266	1.086-1.475	0.0028
Brain—spinal cord (cervical c-1)	5	116	1.198	1.081-1.326	0.0007	9	141	1.295	1.113-1.508	0.0010
Brain—substantia nigra	2	119	1.200	1.084-1.328	0.0005	5	145	1.301	1.121-1.511	0.0007

## Discussion

The use of GWAS data in MR-based approaches has opened up opportunities to assess and define clinically relevant signatures for a diverse spectrum of diseases. Our study supports the role of a person's underlying circadian rhythm in genetic predisposition to neurodegeneration. We found an association of genetically predicted MP trait with AMD. The correlated trait CHR also had a suggestive risk association with AMD. We also found suggestive evidence for a possible association of genetically predicted SS with AD, and INS with ALS. Surprisingly, however, our study found no evidence to support the association between pain and NDDs.

To date, evidence from observational studies has shown a remarkable heterogeneity in the association of different circadian traits with various NDDs. A recent study investigating the incidence of AMD in 108,225 participants observed that patients with INS were 33% more likely to have subsequent AMD (HR 1.33; 95% CI 1.18, 1.48) (34). Previously, an observational study on 57 patients with neovascular AMD and 108 controls found a significantly increased risk of neovascular AMD in patients sleeping <6 h compared to those sleeping 7-8 h (OR 3.29; 95% CI 1.32, 8.27) (35). Another study failed to detect an association with LS in 316 patients with neovascular AMD compared to 500 patients without AMD (36). However, the study did find an association of LS with geographic atrophy, an advanced form of AMD, in 61 individuals (presence of a discrete area of atrophy with a diameter of ≥175 μm). A recent observational study further reported that individuals who take an afternoon nap are 60% less likely to be diagnosed with late AMD (56 with late AMD vs. 1,204 without AMD) (37). As darkness is known to stimulate the secretion of melatonin from the pineal gland, our findings are in agreement with previous studies showing that increased melatonin synthesis could play a protective role in the pathophysiology of AMD (38). However, a recent randomized controlled trial (RCT) failed to show any beneficial effect of low-level night-time light therapy on the progression of AMD (39).

In contrast to previously reported findings from epidemiological studies, we failed to observe any association of INS, SS, and LS with AMD using the genetic data in this study. However, we observed that MP is more likely to be predisposed to AMD (OR 1.19; 95% CI 1.08, 1.32). Our study suggests that more prolonged exposure to daylight in such individuals could increase the risk for AMD. Our findings are in contrast to a recent meta-analysis of observational studies demonstrating the absence of an association between sunlight exposure and AMD (OR 1.12; 95% CI 0.76, 1.67) (40). One of the possible reasons for this discrepancy could be that only one of the 14 studies included in the meta-analysis was a cohort study. The only included cohort study was a 10-year follow-up study, which demonstrated that participants exposed to summer sun for more than 5 h a day were more likely to show increased retinal pigment (RR 2.99; 95% CI 1.18, 7.6) and develop early age-related maculopathy (RR 2.2; 95% CI 1.02, 4.73) in comparison to those exposed for <2 h per day (41). It has also been suggested that excessive light exposure may induce phototoxic damage to the retinal pigmental epithelium and possibly contribute to the gradual worsening of vision in AMD (42–44).

Compared to the impact of circadian rhythms on other NDDs, the role of sleep-related traits has been well-investigated in AD but with mixed findings. Previous studies have predominantly focused on sleep-wake rhythmicity, showing higher incidence of sleep fragmentations and lower amplitude of circadian rhythmicity in patients with moderate or severe AD (1). Concerning SD, both LS and SS have been previously shown to be linked with the risk of dementia (8, 45, 46). A 17-year longitudinal study investigating sleep characteristics in 11, 247 old-aged Swedish individuals (> 65 years at baseline) observed an association of short (≤ 6 h) and extended (> 9 h) time in bed with a higher incidence of dementia (HR 1.4, 95% CI 1.06, 1.85; HR 1.11, 95% CI 1, 1.24) (8). Our results are in agreement with a previously published study (8). Indeed, we observed a strong causal role of SS in predisposition to AD (OR 1.26; 95% CI 1.08, 1.46). However, our results need to be treated with caution, as the association was lost after excluding the overlapping UKB samples from the AD data set, as demonstrated previously (18). It is also possible that the association was lost because of decrease in sample size, necessitating replication with larger AD data sets in the future.

Sleep disturbances are also frequently observed in patients with ALS. Our MR analysis also suggested a possible causal role of INS in ALS (OR 1.55; 95% CI 1.12, 2.14). A previous observational study has demonstrated decreased sleep efficiency and fragmented sleep architecture in 59 patients with ALS (47). Another study reported the presence of sleep disturbances in more than 2/3 of 40 patients with ALS. The study further reported a diagnosis of INS in 65% of the patients (48). These results are in agreement with a previous study reporting a significantly higher prevalence of INS in 90 patients with motor neuron disease compared to 96 healthy controls (48.9 vs. 31.3%, *p* = 0.014) (49). In summary, reports of sleep disturbance among patients with ALS in small sample-sized observational studies and the suggestive causal role of INS in ALS in this study necessitate a need for conducting large-scale epidemiological studies.

Despite the consistent findings of excessive daytime sleepiness or altered sleep timing in patients with PD, our MR findings demonstrate the absence of any causal role of sleep-related traits in predisposition to PD (1). One possible explanation could be that dopaminergic treatment might have influenced the sleeping behavior of patients with PD, as excessive daytime sleepiness is known to be one of the common side effects of dopaminergic treatment. In such a scenario, causal analysis using biological markers of circadian rhythms such as core body temperature, cortisol, and melatonin rhythms, might potentially shed light on the true relationship between sleep-related traits and PD.

We also failed to observe any causal association of sleep-related traits with MS, although sleep disturbance is a common symptom of MS (1). It is suggested that the sleep disorders observed in patients with MS could be a secondary cause of fatigue, a symptom that affects 9 of 10 patients with MS (50).

Among all NDDs, high prevalence of pain has been observed in patients with AD and PD (1). Assessment of pain in such patients of is often challenging because of associated cognitive and motor impairments (51). Nevertheless, the use of genetic instruments of pain on a general population shows that MCP does not play any causal role in AD and PD. A recent cross-sectional study investigating pain in 100 patients with PD patients showed that pain is more prevalent in patients with advanced-stage PD than patients with early-stage PD suggesting pain to be a consequence of the disease rather than a cause (52). Moreover, pain is a broad concept, and inconsistencies in the measurement of number of available pain behavior rating scales often limit their application in clinical settings.

Our study has several strengths and limitations. We adopted a highly comprehensive approach involving the exploration of several sleep-related traits and pain with commonly prevalent NDD. We further employed multiple MR methods and heterogeneity and sensitivity analysis approaches to confirm the reliability of the observed associations. Concerning limitations, previous observational studies have shown that the impact of sleep and pain-related traits may be dependent on the stage of neurodegeneration or severity of an NDD (2). However, we could not conduct such a stratified analysis because of the non-availability of an individual-level data set for respective NDD. Furthermore, pain is a highly complex trait, and the lack of genetic instruments specific for neuropathic and nociceptive pain may undermine the findings of this study. The possibility of nociceptive pain confounding the causal relationship between neuropathic pain and neurodegeneration cannot be ruled out. One critical assumption for MR is that the effect of a genetic instrument for the main exposure on disease outcome is mediated by its influence on the intermediate trait. As genetic variants associated with sleep (duration or pattern) are highly correlated with pain and other sleep-related traits (duration or pattern), we addressed the potential pleiotropic effect by conducting a multivariable analysis. Our findings of causal association between sleep pattern (CHR or MP) and AMD remained robust after adjusting for the potential pleiotropic effect of SD and pain. However, despite adopting a multivariable MR approach, the possibility of residual confounding due to our inability to simultaneously adjust for all the highly correlated SD-related traits (SD or LS or SS) cannot be ruled out.

Using genetic data, we provide strong evidence that being an MP is a causal risk factor for genetic liability to AMD. There is a necessity for conducting large-scale epidemiological cohort studies to confirm our findings. Additional research is also required to understand the biological pathways underlying these associations, including causal analysis with biochemical makers of sleep and correlated traits associated with sleep.

## Data Availability Statement

The original contributions presented in the study are included in the article/Supplementary Material, further inquiries can be directed to the corresponding author.

## Ethics Statement

Ethical review and approval was not required for the study on human participants in accordance with the local legislation and institutional requirements. Written informed consent from the participants' legal guardian/next of kin was not required to participate in this study in accordance with the national legislation and the institutional requirements.

## Author Contributions

SG designed and conceptualized the study, conducted data extraction, analyzed the data, drafted the manuscript, and revised the final draft. MS supervised the overall study and revised the final draft. Both authors contributed to the article and approved the submitted version.

## Funding

This study was, in part, supported by the EU Joint Programme-Neurodegenerative Diseases Research (JPND) project under the aegis of JPND (www.jpnd.eu) through Germany, BMBF, funding code 01ED1406. MS was further funded by the Michael J. Fox Foundation, USA Genetic Diversity in PD Program: GAP-India Grant ID: 17473 and supported by grants from the German Research Council (DFG/SH 599/6-1 to MS), and MSA Coalition.

## Conflict of Interest

The authors declare that the research was conducted in the absence of any commercial or financial relationships that could be construed as a potential conflict of interest.

## Publisher's Note

All claims expressed in this article are solely those of the authors and do not necessarily represent those of their affiliated organizations, or those of the publisher, the editors and the reviewers. Any product that may be evaluated in this article, or claim that may be made by its manufacturer, is not guaranteed or endorsed by the publisher.
